# Epoxytiglianes potentiate the activity of colistin against resistant *Escherichia coli* via modification of the bacterial cell membrane

**DOI:** 10.1128/mbio.02314-25

**Published:** 2025-12-29

**Authors:** Manon F. Pritchard, Wenya Xue, Jingxiang Wu, Francesca Boardman, Mei Li, Yuqing Zhou, Saira Khan, Lydia C. Powell, Joana Stokniene, Josh Davies-Jones, Philip R. Davies, Niklaas J. Buurma, Georgina E. Menzies, Owen B. Spiller, Timothy R. Walsh, Paul Reddell, Katja E. Hill, David W. Thomas

**Affiliations:** 1 Advanced Therapies Group, School of Dentistry, Cardiff University 70720, Cardiff, United Kingdom; 2 Department of Implantology, School and Hospital of Stomatology, Wenzhou Medical University 26453, Wenzhou, China; 3 Ineos Oxford Institute for Antimicrobial Research, University of Oxford 6396, Oxford, United Kingdom; 4 Microbiology and Infectious Disease Group, Swansea University Medical School, Swansea University 151375, Swansea, United Kingdom; 5 QBiotics Group Ltd., Yungaburra, Australia; 6 School of Chemistry, Cardiff Catalysis Institute, Cardiff University 151286, Cardiff, United Kingdom; 7 Physical Organic Chemistry Centre, School of Chemistry, Cardiff University 151286, Cardiff, United Kingdom; 8 School of Biosciences, Cardiff University 151285, Cardiff, United Kingdom; 9 Medical Microbiology, School of Medicine, Cardiff University, Cardiff, United Kingdom; Prairie View A&M University, Prairie View, Texas, USA

**Keywords:** antimicrobial resistance (AMR), plasmid, *mcr-1*, *mcr-3*

## Abstract

The unrestricted use of colistin in animal husbandry has led to the emergence of mobile plasmid-borne colistin resistance (*mcr*). The antimicrobial epoxytigliane, EBC-1013, has been shown to be effective in the topical treatment of biofilm-mediated infections *in vivo*. Hypothesizing that EBC-1013 interacts with the modified bacterial outer membrane of colistin-resistant *Escherichia coli,* we investigated its ability to potentiate the activity of colistin using fractional inhibitory concentration (FIC) index determinations against environmental *mcr* Enterobacteriaceae. Molecular dynamics (MD) simulations were used to visualize EBC-1013 interactions with the outer membrane *in silico*. Modification of outer membrane surface chemistry and adhesion properties was characterized using X-ray photoelectron spectroscopy (XPS) and hydrophobicity and bacterial membrane permeability assays. Anti-biofilm activity was quantified using confocal laser scanning microscopy (CLSM). Checkerboard assays revealed synergistic effects of EBC-1013 with colistin (FIC ≤ 0.5) in 6/9 strains. MD simulations demonstrated weak EBC-1013 interactions with colistin and the outer membrane. XPS suggested modification of the outer membrane in both colistin-sensitive and colistin-resistant *E. coli,* as well as the interaction of colistin and EBC-1013 with the phosphoethanolamine (pEtN)-substituted *mcr* lipid moiety. EBC-1013 binding significantly decreased bacterial hydrophilicity and potentiated membrane permeability induced by colistin (*P* < 0.05). In the biofilm model, potentiation was also evident, with a marked decrease in bacterial biomass in EBC-1013/colistin combined treatments (*P* < 0.0001) for colistin-resistant *E. coli*. The results highlight the ability of EBC-1013 to modify the bacterial membrane of colistin-resistant *E. coli* and potentiate the activity of colistin in the treatment of multidrug-resistant wound bacteria.

**IMPORTANCE:**

Resistance to colistin, an antibiotic of last resort for hard-to-treat infections, is on the increase. Therefore, the need to develop new antimicrobials to tackle antimicrobial resistance is of paramount importance. The epoxytiglianes represent an exciting range of molecules with a diverse range of biological effects in human and veterinary applications, including antimicrobial properties. In this study, we show how EBC-1013 interacts with the outer surface of colistin-resistant *Escherichia coli* cells, inducing chemical and structural changes to the cell wall, making it susceptible again to colistin treatment. This ability of EBC-1013 to enhance the activity of colistin against a range of colistin-resistant *E. coli* suggests that EBC-1013, alone (or as a combination therapy), has potential as a new treatment strategy to treat antibiotic-resistant bacterial wound infections and reduce antibiotic usage.

This study is registered with ClinicalTrials.gov as (Australian New Zealand Clinical Trials Register: ACTRN12624000544572).

## INTRODUCTION

Colistin plays an important role in the treatment of infections caused by multidrug-resistant (MDR) Gram-negative Enterobacteriaceae. The bactericidal activity of colistin targets the lipid A component of the outer membrane; the amphipathic lipopeptide colistin crossing and effectively destabilizing the membrane (1) and inducing leakage of intracellular contents and cell lysis (2). While inherent colistin resistance exists in certain Gram-negative bacteria, e.g., *Neisseria meningitidis* and *Burkholderia* species, in the last decade, the unrestricted use of colistin (polymyxin E) in animal husbandry has led to the emergence and global dissemination of plasmid-borne (*mcr-1*) colistin resistance (3).

To date, >10 mobile colistin resistance (*mcr*) gene determinants have been identified encoding a phosphoethanolamine (pEtN) transferase which modifies the polycationic colistin target, catalyzing transfer of pEtN onto the anionic lipid A moiety of lipopolysaccharide (LPS) in the bacterial outer membrane (3–6). The reduction in negative charge results in decreased colistin binding to pEtN-modified lipid A and impaired activity. Phylogenetic analysis has revealed that different *mcr* genes are evolutionarily distinct (1); *mcr-3* sharing only 45% nucleotide identity with *mcr-1* (7). Growth competition models have shown that expression of *mcr-3* represents a lower fitness cost than *mcr-1,* which is associated with higher stability of plasmid carriage in parental *Escherichia coli* strains (8). However, *mcr-1* remains the most prevalent in Enterobacteriaceae isolated from humans (9).

To improve the effectiveness of colistin (which is dose-limited due to nephrotoxicity), combination therapies have been evaluated. These include drug repurposing and targeting of enzymes responsible for lipid A modification (10) and lipid synthesis (11) to disrupt the cytoplasmic membrane and exploiting cross-sensitivity with other antimicrobials (e.g., amikacin, clarithromycin, minocycline). All have been shown to enhance the activity of colistin against *mcr-1* harboring strains (11–13), with synergy between hydrophobic molecules and polymyxins reported to be more pronounced than with hydrophilic molecules (13–15).

Exploitation of the plant kingdom is an increasing resource for antibacterial drug discovery (16). In response to bacteria (and viruses), plants have evolved complex ways to resist invasion, effectively compartmentalizing disease and inactivating pathogens via sophisticated, multi-component mechanisms, including the use of RNA interference to resist pathogen virulence and anti-silencing suppressors as counter-defensive measures to prevent pathogen-induced RNA silencing (17, 18). A large number of phytochemicals, including phenolic compounds, terpenoids, and alkaloids, have been proposed as antimicrobial candidates (due to their activity and low levels of resistance) (19, 20) and in combination therapies may increase antibiotic activity (21–23). Recently, the antimicrobial activity of a semi-synthetic diterpene ester with C12 and C13 dihexanoate ester chains (EBC-1013), derived from the Queensland blushwood tree (*Fontainea picrosperma*), has been demonstrated *in vitro* (24). In addition to modifying pseudomonal virulence factor (pyocyanin) production and inducing biofilm disruption (unlike the less lipophilic, naturally occurring EBC-147 with C12 propanoate and C13 methylbutyrate esters), topically administered EBC-1013 was also shown *in vivo* to induce innate immune system resolution of infection in burns and diabetic wounds (24). We proposed that these effects result from interaction with the Gram-negative outer membrane.

Antimicrobials targeting the outer membrane and membrane assembly represent attractive candidates as antibiotic adjuvants (25, 26). The effect of colistin on both the sensitive and resistant bacterial membranes has been well documented (27). Interestingly, *mcr-1* confers little resistance to colistin-induced disruption of the outer membrane generated by the hydrophobic colistin fatty-acyl tail (28), but impairs uptake into the cell, effectively blocking cell lysis (15). We hypothesized that the antimicrobial activity of EBC-1013 reflected its lipophilic (C12 and C13 ester) side-chain interaction with the lipid A-modified bacterial outer membrane. Here, we studied the ability of EBC-1013 to potentiate the activity of colistin against a panel of colistin-resistant (*mcr*) Gram-negative bacteria, comparing transconjugants in a common *E. coli* host (farmyard isolate CX-17) with environmental (farmyard) isolates and mapped its binding and chemical modification of the outer membrane. EBC-1013 induced changes in bacterial hydrophobicity and outer membrane integrity that were evident at the nanoscale, as was synergistic potentiation of colistin against colistin-resistant *E. coli* in both planktonic and biofilm colistin-resistant bacterial models.

## RESULTS

### EBC-1013 induces potentiation of colistin against *mcr* Enterobacteriaceae

A panel of nine colistin-sensitive*, mcr-1* or *mcr-3* harboring environmental Enterobacteriaceae (Table S1; with minimum inhibitory concentration [MIC] to colistin ranging between 0.064 to 4 µg/mL) was employed to test potentiation of colistin using a checkerboard assay (Table 1). Against these strains, EBC-1013 (and EBC-147) had MICs >512 µg/mL. Despite this, however, EBC-1013 demonstrated marked synergistic effects against 5/8 *mcr* strains tested (as well as the *E. coli* CX-17 plasmid-free recipient strain) in combination with colistin (fractional inhibitory concentration [FIC] indices ≤ 0.5). This synergy was evident at concentrations of EBC-1013 ≤32 μg/mL. Taking the lowest effective EBC-1013 dose in the combination treatment, potentiation of colistin was associated with a fourfold to sixfold decrease in MIC (from >512 ug/mL to 16–64 µg/mL; Table 1). No significant interactions between EBC-147 and colistin were noted.

**TABLE 1:** MIC, FIC index determinations, and best (lowest effective) combination dose for epoxytiglianes^*a*^ and colistin against colistin-sensitive and *mcr* Enterobacteriaceae

	MIC	FIC index^*b*^		Best (lowest effective) combination dose^*c*^	
Strains	Colistin	Colistin + EBC-1013	Colistin + EBC-147	Colistin	EBC-1013
CX-17	0.064	**0.266**	1.016	0.008	32
CX-17(pPN16) *mcr*-1	4	**0.313**	1.016	1	64
CX-17(pT145.4) *mcr*-1	2	0.625	1.008	1	16
CX-17(pWJ1) *mcr-3*	2	**0.375**	1.031	0.5	32
CX-17(pT145.8) *mcr-3*	4	**0.375**	1.016	1	16
HRS.18 *mcr-1.1*	4	**0.375**	0.563	1	16
HRS.1821 *mcr-3.20*	4	0.563	1.031	1	256
FMM.1860 *mcr-3.21*	4	**0.375**	1.008	1	16
NNMR 49.b *mcr-1*	4	0.508	1.056	0.5	256

^*a*^ MICs (µg/mL) for EBC-1013 and EBC-147 for all strains were >512 µg/mL.

^*b*^ FIC index values were interpreted as follows: synergy (≤0.5), indifference (>0.5 to <2), and antagonism (≥2); values in bold show apparent synergy.

^*c*^ Best (lowest effective) dose (µg/mL) for the combination colistin and EBC-1013 treatment.

### EBC-1013 and colistin interact at the *E. coli* outer membrane

**Fig 1:**
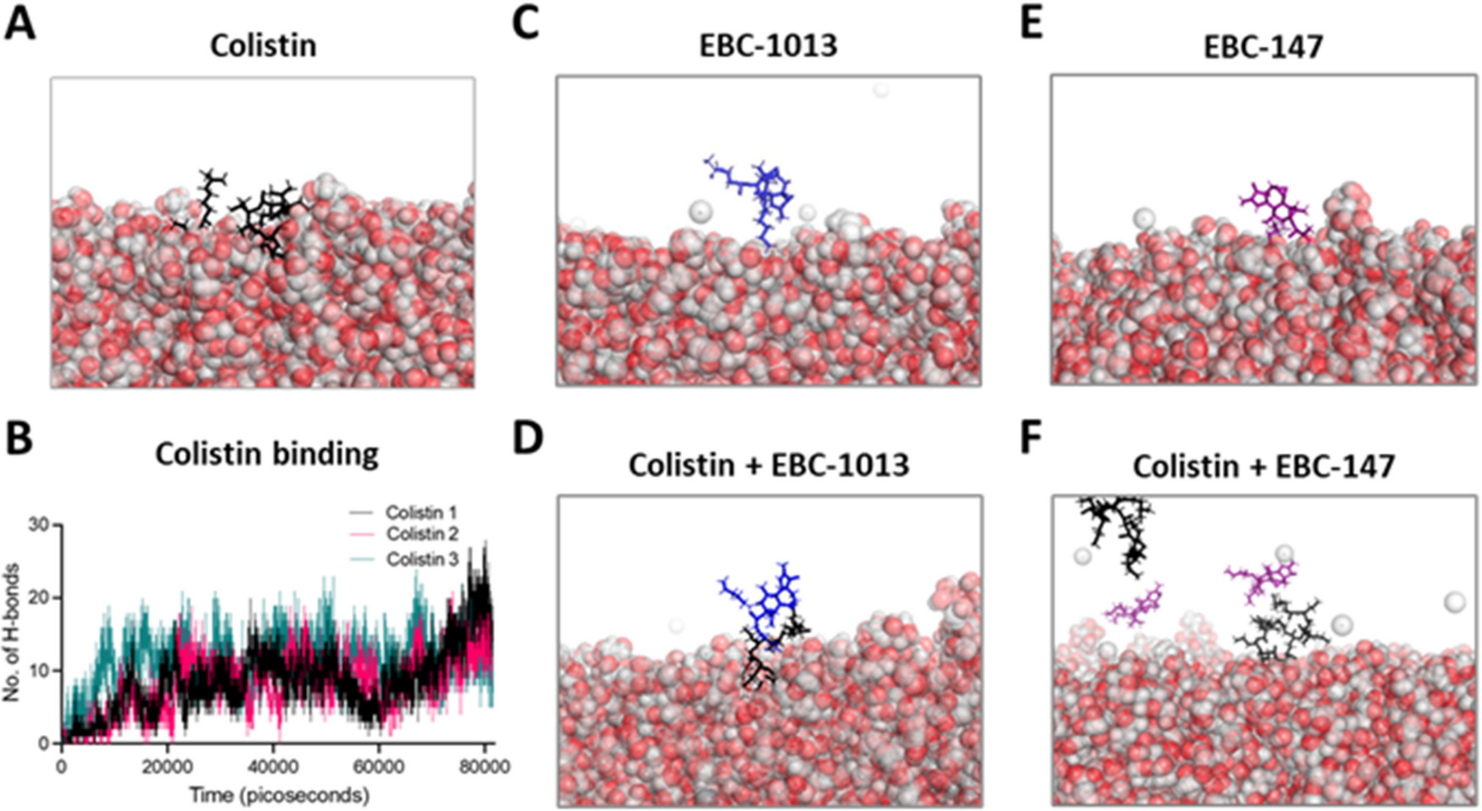
MD simulations of (**A**) colistin (black) and (**B**) associated hydrogen bonding with adjacent sugar molecules between colistin and *E. coli* over time (with *n* = 3 different colistin molecules represented by the different lines), (**C**) EBC-1013 (blue), (**D**) EBC-1013 (blue) with colistin, (**E**) EBC-147 (purple), (**F**) EBC-147 (purple) with colistin with an *E. coli* membrane. Images were produced using PyMOL and are snapshots taken from the first 50 n.s. of the simulation time.

Molecular dynamics (MD) simulations revealed that colistin consistently became embedded in the outer membrane (Fig. 1A), disrupting the surface, forming multiple hydrogen (H) bonds with adjacent sugar molecules in all repeat simulations (Fig. 1B); all colistin molecules eventually becoming embedded in the membrane. EBC-1013 alone formed unsustained H-bonds, typified by rapid attachment and detachment of EBC-1013 from the cell surface (Fig. 1C) and remained unbound at the end of the MD simulation (Fig. S1). The inclusion of EBC-1013 and colistin in the same simulation was associated with periodic co-localization (Fig. 1D), with H-bonds also forming between the two molecules within the outer membrane. However, at the end of the MD simulation, only colistin remained embedded in the outer membrane (Fig. S1). Although EBC-147 also demonstrated formation of unsustained H-bonds with the outer membrane (Fig. 1E), it was to a lesser extent than seen with EBC-1013 (a total of 971 hydrogen bonds formed compared to 2,214, respectively, during the entire simulation), and the EBC-147 molecules remained away from the outer membrane for most of the simulation. Interactions between colistin and EBC-147 also occurred, but to a lesser extent (a total of 99 hydrogen bonds formed compared to 151, respectively) (Fig. 1F), with colistin molecules becoming embedded in the outer membrane in 2/3 simulations (Fig. S1).

### EBC-1013 induces modification of the outer membrane surface chemistry of colistin-resistant *E. coli*

**Fig 2:**
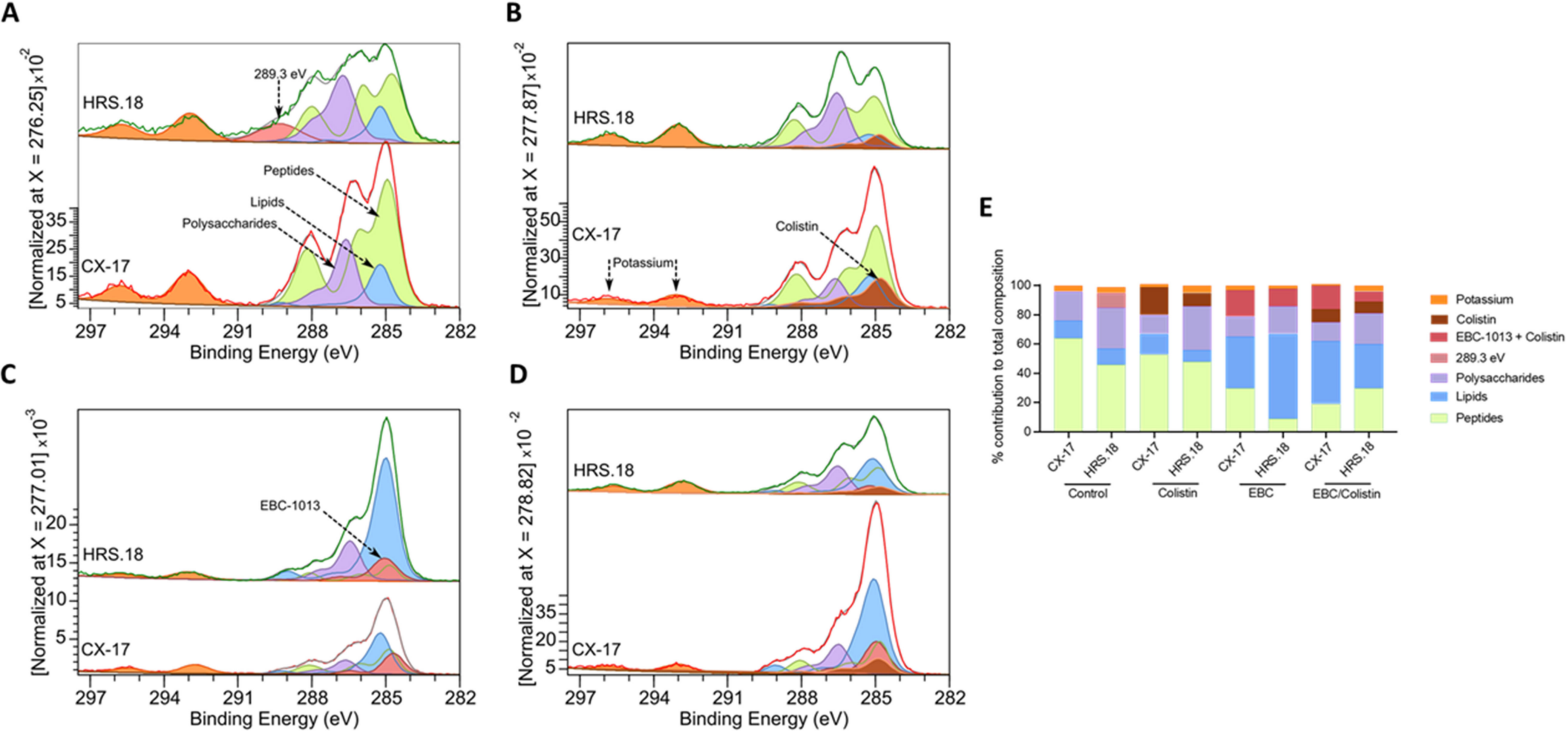
XPS screening. Deconvolution of the C(1s) region of XP spectra of HRS.18 (colistin-resistant) and CX-17 (colistin-sensitive) *E. coli* treated as follows: (**A**) untreated control, (**B**) colistin only, (**C**) EBC-1013 only, (**D**) colistin and EBC-1013, (**E**) calculated % contribution of each component (surface composition) to the total in each sample. HRS.18 (the resistant strain) has an extra carbon species at 289.3 eV contributing ~10% concentration.

The effects of colistin and EBC-1013 on the outer bacterial cell membrane were studied using X-ray photoelectron spectroscopy (XPS), treated for 1 h, providing a quantitative description of the membrane in terms of the relative presence of different elements with distinct binding energies (up to an approximate depth of 10 nm). Deconvoluted data in the C(1s) region reflects contributions from lipids, peptides, and polysaccharides, with additional terms for colistin and EBC-1013. While the XPS data are quantitative, deconvolution of the data in terms of these five components provides a qualitative description of changes in chemical composition of the outer membrane of colistin-sensitive CX-17 and *mcr* HRS.18 *E. coli* (Fig. 2).

The spectra for both strains were very similar, save for the presence in HRS.18 of an additional C(1s) peak at 289.3 eV (Fig. 2A), a N(1s) peak at 402.3 eV which is typical of NH_4_^+^; a P(2p) peak at ~135 eV; and an additional O(1s) peak at ~535 eV. These four additional peaks reflect the known addition of pEtN to the hepta-acylated form of lipid A in the resistant strain. The deconvoluted C(1s) XPS spectra of colistin-sensitive CX-17 and *mcr* HRS.18 *E. coli* following incubation with colistin demonstrated clear differences between the two strains. Colistin became clearly bound to the CX-17 lipopolysaccharide, which was less prominent in the *mcr* HRS.18 strain (Fig. 2B). The addition of 128 μg/mL EBC-1013 alone induced marked increases in the lipid signal at the surface of the *mcr* HRS.18 bacterial outer membrane, whilst a reduction was seen in CX-17 (Fig. 2C). Combination treatment also showed an increase in the lipid proportion relative to the control, as well as signals for colistin and EBC-1013 in both the *mcr* HRS.18 strain and in CX-17 (Fig. 2D). In all cases, the presence of EBC-1013 markedly increased the visibility of the lipid fraction in both strains (Fig. 2E). The incorporation or attachment of both colistin and EBC-1013 to the bacterial outer membrane was also evident in both strains through suppression of the relative P(2p) signal by both compounds and the general suppression of N(1s), P(2p), and S(2p) signals by EBC-1013 (Table S2). The additional features present in the N(1s), O(1s), and P(2p) regions of the resistant strain (attributed to the ethanolamine modification of the lipid) were not apparent upon treatment with either colistin or EBC-1013 or in combination.

### EBC-1013 binding induces decreases in the hydrophilicity of colistin-resistant *E. coli*

**Fig 3:**
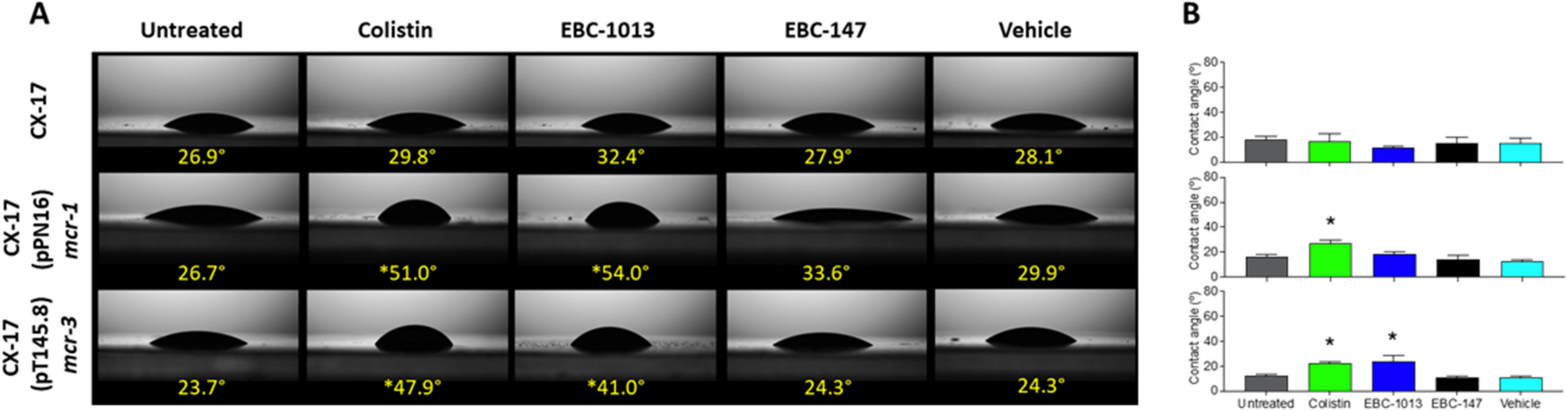
Determination of hydrophobicity. Contact angle (°) measurements (**A**) at the 0 time point and (**B**) mean contact angle over 0–10 s for *E. coli* bacterial lawns treated with epoxytiglianes (256 μg/mL) alongside untreated, colistin (½ MIC; 0.032 or 2 μg/mL) and vehicle (256 μg/mL equivalent) controls. Contact angle measurements of <90° are indicative of a hydrophilic surface. *Significantly different from untreated control (*P* < 0.05).

Surface contact angle (SCA) measurements demonstrated that those for colistin-sensitive *E. coli* CX-17 remained hydrophilic and unchanged (<35°) following treatment with colistin (½ MIC; 0.032 μg/mL), epoxytigliane (EBC-1013; 256 µg/mL), or vehicle-equivalent controls (256 µg/mL). In contrast, treatment of *mcr* strains CX-17(pPN16), CX-17(pT145.8), HRS.18, and HRS.1821 with colistin (½ MIC; 2 μg/mL) or EBC-1013 (256 μg/mL) resulted in markedly increased SCAs (>40°) of the bacterial lawns, becoming less hydrophilic; being significant with EBC-1013 for all four strains and with colistin for 3/4 strains tested (Fig. 3A; Fig. S2A; *P* < 0.05). EBC-1013 and colistin-treated bacterial lawns also demonstrated increased mean cell surface hydrophobicity over the whole 0–10 s time period of the experiment for 3/4 strains tested (Fig. 3B; Fig. S2B; *P* < 0.05). In these experiments, EBC-147 and the vehicle control had no apparent effect on bacterial hydrophobicity.

### EBC-1013 treatment potentiates membrane permeabilization of *E. coli* by colistin

**Fig 4:**
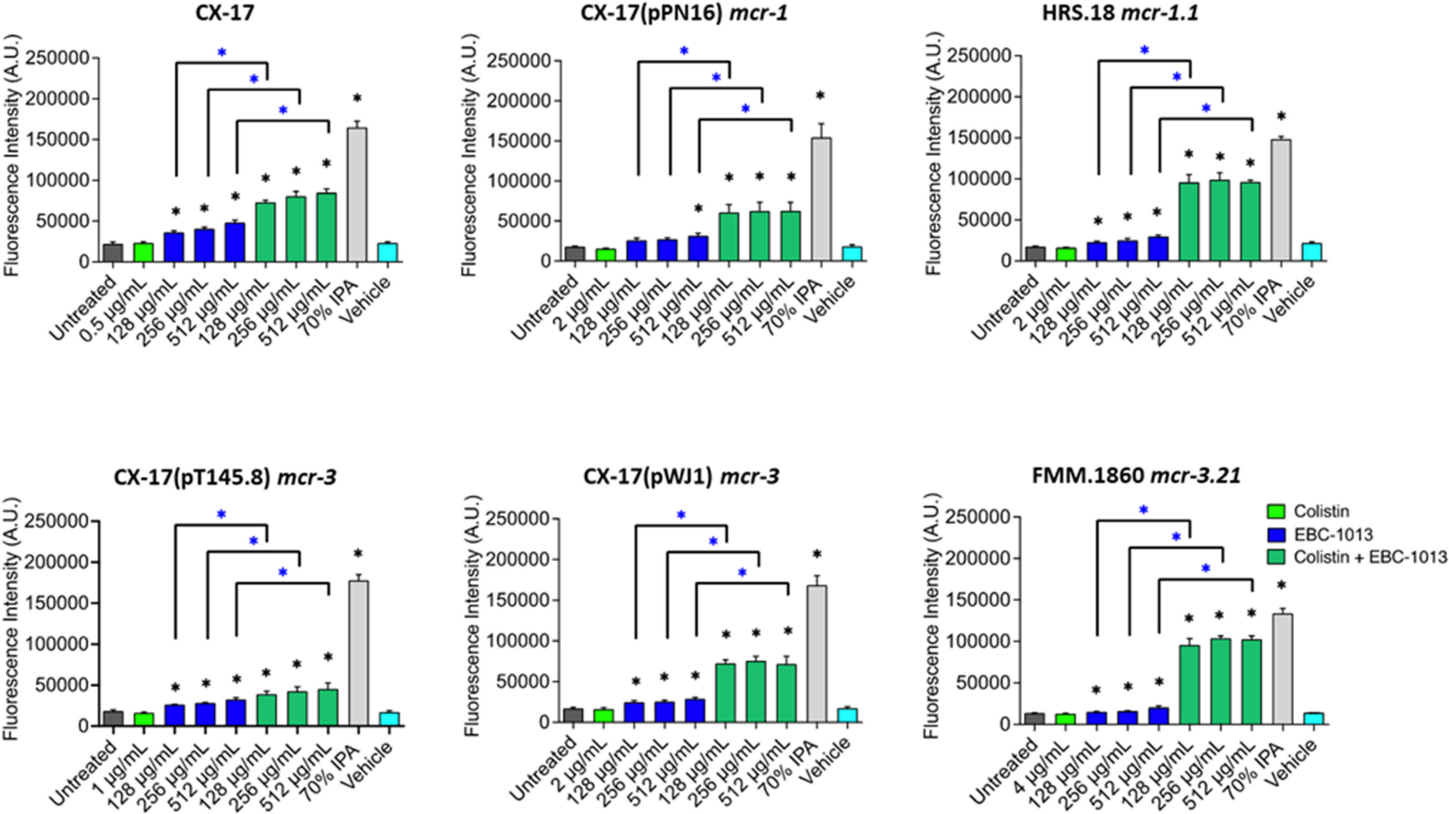
Cell permeabilization assay showing the effects of colistin ± EBC-1013 treatment on colistin-sensitive (CX-17) and colistin-resistant *E. coli*. Colistin (used at the same dilution just below efficacy for each strain; see Fig. S3), EBC-1013 (128, 256, 512 μg/mL), vehicle equivalent controls (ethanol; 512 μg/mL), and 70% isopropanol (IPA) positive controls were included. Results are expressed as fluorescence intensity (A.U.). *Represents significantly different compared to the untreated control (black asterisk) ; *represents significantly different compared to the equivalent EBC-1013 (no colistin) control (*n* = 3; *P* < 0.05) (blue asterisk).

**Fig 5:**
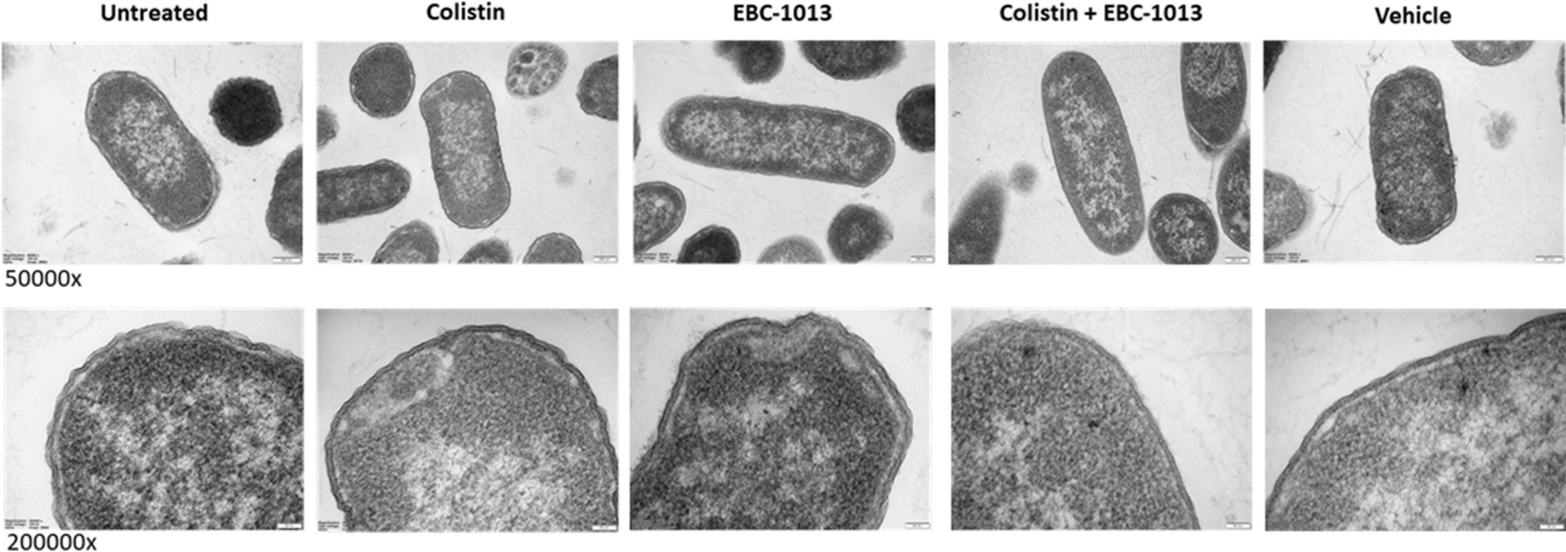
TEM of *E. coli* HRS.18 (*mcr-1.1*) following treatment with colistin (2 μg/mL) and/or EBC-1013 (128 μg/mL) alongside ethanol vehicle (128 μg/mL) and untreated (phosphate-buffered saline [PBS]) controls (magnification as shown).

A permeabilization assay was used to investigate whether combination therapy could lower the effective dose of colistin required to disrupt the integrity of the cell membrane of *mcr*-harboring pathogens. EBC-1013 alone at 128 μg/mL exhibited dose-dependent cell permeabilization when compared to the untreated control (Fig. 4; *P* < 0.05); the exception being *E. coli* CX-17(pPN16) *mcr-1,* in which only the highest EBC-1013 concentration (512 μg/mL) induced significant permeabilization. The lowest effective concentration of colistin was confirmed prior to conducting combination therapy experiments, with colistin-sensitive CX-17 permeabilized at 1 μg/mL, while for the colistin-resistant strains, permeabilization occurred between 2 and 8 μg/mL (Fig. S3) equating to MIC or 2× MIC. Combination treatments using colistin (½ MIC) and EBC-1013 showed significant permeabilization (at <128 µg/mL EBC-1013) compared with untreated and EBC-1013-only controls (*P* < 0.05). Treatment with vehicle controls (512 μg/mL) failed to affect permeabilization of the bacteria. Transmission electron microscopy (TEM) of colistin-resistant *E. coli* (HRS.18) treated with EBC-1013 and the combination treatment demonstrated structural rearrangement of the outer membrane (noted by cell debris outside the cell wall; Fig. 5), which was absent in the colistin-only and vehicle-only treated controls.

### EBC-1013 enhances biofilm disruption by colistin against colistin-resistant *E. coli* in established biofilms

**Fig 6:**
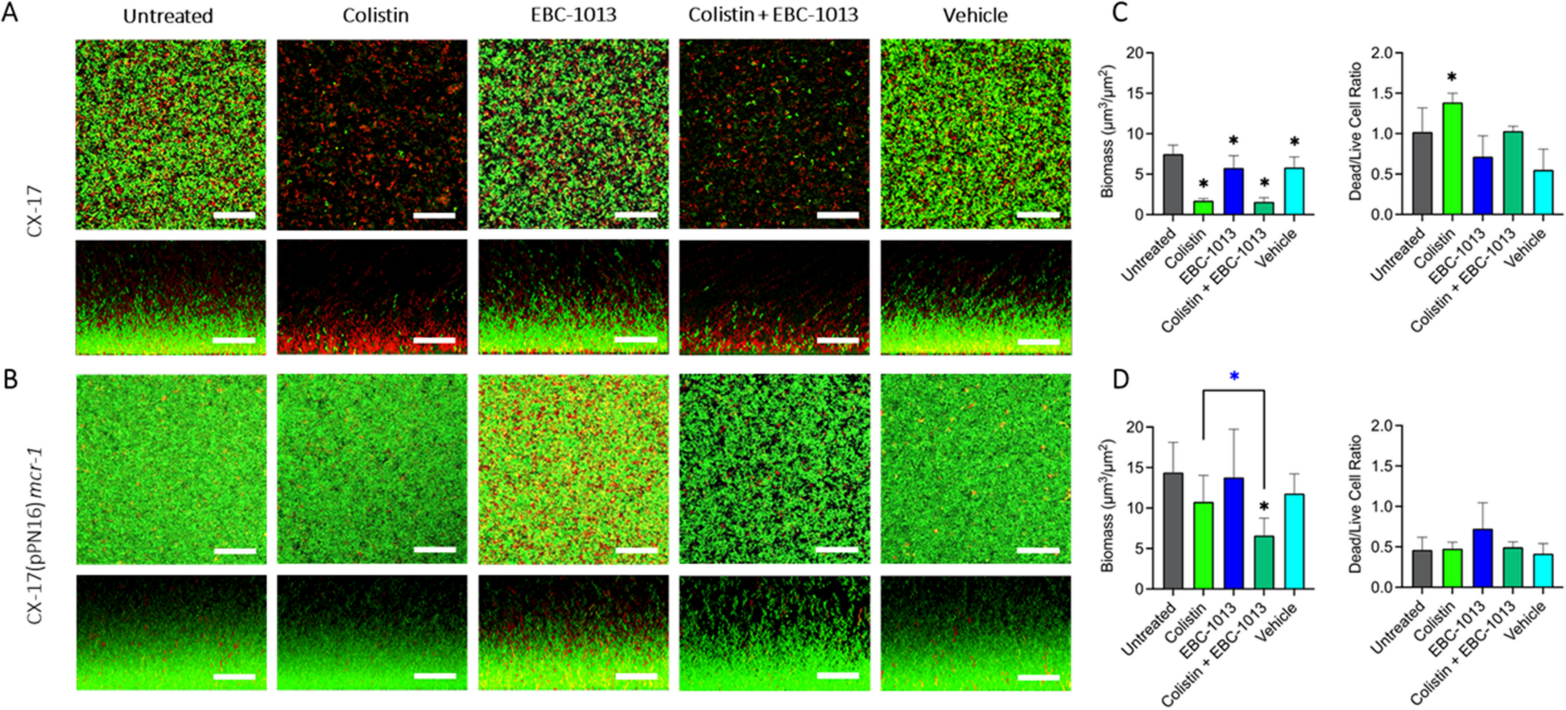
CLSM with LIVE/DEAD staining (aerial and side views) showing disruption of 18 h established biofilms treated (24 h) with colistin [8 μg/mL for CX-17 and 32 μg/mL for CX-17(pPN16)], EBC-1013 (128 μg/mL) or combined therapy, alongside untreated and vehicle equivalent (ethanol) controls. (**A**) Colistin-sensitive *E. coli* CX-17. (**B**) Colistin-resistant *E. coli* CX-17(pPN16) *mcr-1* (scale bar = 30 μm). (**C, D**) Corresponding COMSTAT image analysis of biofilm CLSM z-stack images. *Represents significantly different compared to the untreated control (black asterisk) ; *represents significant difference between colistin and the combination colistin and EBC-1013 therapy (*n* = 3; *P* < 0.05) (blue asterisk) .

The effect of EBC-1013, colistin, and combination treatment was tested on established *E. coli* (18 h) biofilms using confocal laser scanning microscopy (CLSM; Fig. 6). Colistin-sensitive *E. coli* CX-17 strain formed a less robust and less viable biofilm structure compared to the colistin-resistant *E. coli* CX-17(pPN16) variant (Fig. 6A and B). However, as expected, COMSTAT image analysis demonstrated significant reductions in colistin-sensitive CX-17 biofilm biomass (*P* < 0.0001) and increases in DEAD/LIVE bacterial cell ratio (*P* < 0.05) for colistin-only treatment in comparison to the untreated and vehicle controls (Fig. 6A and B; Table S3). EBC-1013 treatment of the CX-17 biofilms also resulted in a decrease in biomass, although to a much lesser extent in comparison to the untreated control (*P* < 0.05); however, this effect was not apparent when compared to the vehicle control. In contrast, both individual colistin and EBC-1013 treatments failed to significantly reduce the biofilm biomass of colistin-resistant *E. coli* CX-17(pPN16) (*P* > 0.05). While EBC-1013 treatment led to an increase in the number of dead cells compared to untreated and vehicle control groups, this was not statistically significant (*P* > 0.05; dead/live cell ratio). The combination treatment of EBC-1013 and colistin, however, resulted in significant reductions in biomass of colistin-resistant CX-17(pPN16) biofilms (*P* < 0.01) in comparison to the colistin, EBC-1013, untreated, and vehicle controls (Fig. 6C and D).

## DISCUSSION

The majority of bacterial pathogens on the World Health Organization priority list are Gram-negative (29); the outer membrane of Gram-negative bacteria and its associated components representing a primary, dynamic macromolecular barrier to antimicrobial therapies. Targeting the function and integrity of the membrane has been used to induce cross-sensitivity to co-administered antimicrobial agents (30, 31). However, the importance of the membrane in maintaining cell viability means that acquired resistance mechanisms that modify the lipid membrane (e.g., *mcr* carriage) are often associated with distinct fitness costs (32).

Based on our previous MD simulations in *Pseudomonas aeruginosa* (24), we hypothesized that the hydrophobic C12 and C13 esters in EBC-1013 would facilitate interaction of the molecule with the *E. coli* outer membrane in the presence of colistin. As previously predicted by reference 33 in MD simulations, interaction of the cationic peptide colistin with the outer membrane of colistin-sensitive *E. coli* developed rapidly, leading to the release of divalent cations (especially calcium) and disruption of the membrane. This hypothesis was confirmed here, although the simulated interactions of EBC-1013 with the membrane alone were relatively weak; a finding in keeping with the low intrinsic antimicrobial activity of the compound (MIC > 512 µg/mL; [24]). In the presence of colistin, however, the surface interaction of the epoxytigliane molecule differed markedly, with EBC-1013 becoming embedded and co-localized with colistin within the membrane. In contrast, EBC-147 (a C12 propanoate and C13 methylbutyrate ester), which exhibits a reduced interaction with the cell membrane (24), was shown to interact with colistin already bound to the surface, with far fewer direct interactions with the outer membrane. We speculate that the co-localization of EBC-1013 and colistin near the membrane surface helps to disrupt a greater number of the surface interactions, allowing calcium release and degradation of membrane stability.

The lipid outer membrane of Gram-negative bacteria is a dynamic structure comprised of phospholipids, lipoproteins, LPS, and porins. XPS (with a penetration depth of <10 nm) was employed to characterize the effects of EBC-1013 on the cell surface chemistry of colistin-sensitive and colistin-resistant *E. coli*. XPS was able to differentiate between the strains tested, with *mcr* carriage resulting in unique peaks in the C(1s), P(2p), and N(1s) regions of the spectrum denoting lipid A substitution with pEtN (34). Significantly, the signals attributed to pEtN modification disappeared following treatment with colistin and EBC-1013 (or both), probably due to blanketing and obscuring by the treatments, moving pEtN out of range of XPS detection. Deconvoluted XPS data showed that both colistin and EBC-1013 interact with the membrane, but that the interaction of EBC-1013 appeared to be much weaker than that of colistin. This was evident despite the significantly higher overall concentrations of EBC-1013 used (128 μg/mL), which yielded similar amounts of membrane-bound material to that of colistin (0.5 or 2 μg/mL). XPS also demonstrated increased visibility of the lipids upon exposure to EBC-1013 and EBC-1013/colistin co-treatment during the 1 h incubation period. These observations suggest that outer membrane disruption occurs on a timescale significantly longer than accessible in the MD simulations. The rapidity of treatment response to EBC-1013 treatment observed here does not support EptA inhibition. While these observations support the direct interaction between pETN and EBC-1013, further *in vitro* work into the interactions between EBC-1013 and the pETN-substituted LPS is ongoing to determine the direct molecular mechanism of action. Interestingly, numerous studies have described terpenes in the potentiation of antibiotics, with a range of mechanisms proposed, including increases in outer membrane lipid release (35) and as efflux-pump inhibitors (36, 37).

Outer membrane hydrophobicity is a key determinant of bacterial virulence, coaggregation, and adhesion (38). To determine the impact of the observed surface chemistry on the bacterial outer membrane, we studied the physicochemical effects of EBC-1013 on colistin-sensitive and colistin-resistant *E. coli*. While neither EBC-1013 nor colistin affected the hydrophobicity of the host CX-17 strain, in colistin-resistant *E. coli,* both colistin and EBC-1013 increased hydrophobicity. In keeping with the MD simulations, EBC-147 had no effect on the hydrophobicity of any of the strains. These changes in cellular hydrophobicity of the bacteria following EBC treatment suggested chemical alterations in the lipid outer membrane, which have been described with other phytochemicals (39, 40). In lipid homeostasis, epoxytiglianes, such as EBC-1013, have also been proposed to act as diacylglycerol (DAG) mimetics (41). DAGs are important components of the bacterial lipid membrane and are known to act as lipid secondary messengers (at least in eukaryotic cells). DAG accumulation has previously been shown to inhibit lipid A pEtN transferase (EptA)-induced modification in resistant bacteria, restore cell envelope homeostasis, and facilitate the loss of antibiotic resistance (42).

The observed modification of the bacterial outer membrane induced by colistin treatment was mirrored in the effects of EBC-1013 on cellular permeability (inner membrane perforation) in a range of colistin-resistant *E. coli*. Whilst colistin (at ≤½ MIC) induced no permeability of any strain tested, EBC-1013 (at 128 µg/mL) induced significant membrane permeabilization in all strains. Most striking, however, was the synergy between EBC-1013 and colistin against all strains (including the sensitive CX-17 and five *mcr* strains). EBC-1013 induced increased bacterial membrane permeability, which was in marked contrast to colistin alone, which (even at 4 µg/mL) failed to induce permeability in the *mcr* strains. A number of agents targeting the membrane integrity of bacteria (from phytochemicals to antimicrobial peptides) have been proposed to potentiate antimicrobial activity (30), including that of colistin against MDR Gram-negative pathogenic bacteria, such as *E. coli* (43). Indeed, other terpene-based antimicrobial agents have been suggested to directly inhibit growth and efflux pump activity and can also induce cell membrane disruption and antimicrobial activity (44). Similarly, the level of permeability induced by EBC-1013 alone was markedly lower than that observed in the combination therapies. Here, enhanced permeability of the inner membrane induced by the combination treatment across a range of *mcr* isolates concurs with modification of the outer membrane-LPS evident in the XPS.

It has been hypothesized that lipid changes induced by *mcr* may affect the susceptibility of bacteria to lipophilic, hydrophobic agents, such as EBC-1013 (24). Permeability studies have shown that a *Δmcr-1* deletion mutant of pHNSHP45 demonstrated decreased resistance to colistin, resulting in a significant change in cell permeability (33). Interestingly, the acyclic terpene alcohol farnesol has also been shown to enhance cell permeabilization in combination with colistin against colistin-resistant *E. coli* (45).

Potentiation of colistin was evident in 5/8 of the *mcr* strains tested, being associated with a fourfold to sixfold decrease in MIC (lowest effective EBC-1013 dose in combination; from >512 ug/mL to 16–64 µg/mL). In keeping with the hypothesis that the observed potentiation by EBC-1013 is mediated by interaction with the outer membrane, MD simulations demonstrated that EBC-147 (which exhibited minimal interactions in the model) did not demonstrate potentiation of colistin against any of the *mcr* isolates.

While the outer membrane effects of EBC-1013 in mediating the potentiation of colistin were evident in planktonic culture (i.e., MICs), bacterial populations in health and disease predominantly exist in biofilms (46). Within bacterial biofilms, the complex, charged polymer matrix of the extracellular polymeric substance represents an important barrier to diffusion and effectiveness of antimicrobial therapies (47). However, biofilm exposure to sub-inhibitory concentrations of antibiotics can induce eDNA release and increase biofilm formation as a proposed stress response (48). We have previously reported the ability of the hydrophobic EBC-1013 to effectively disrupt the established biofilm matrix of *E. coli* (evidenced by increased nanoparticle diffusion and direct imaging) and dysregulate quorum-sensing (QS)-regulated motility and virulence (24). In this study, colistin induced significant changes to biofilm biomass in colistin-sensitive bacterial biofilms. This contrasted markedly with the effects on pEtN-modified *mcr* biofilms, in which colistin was ineffective. The potentiation of sub-inhibitory concentrations of colistin by EBC-1013 in the biofilm model here is indicative of both outer/inner membrane effects and also destabilization of the biofilm matrix induced by EBC-1013 in the colistin-resistant *E. coli* biofilms (49).

Numerous strategies have been proposed to potentiate the activity of conventional antibiotics against MDR Gram-negative bacteria, including combination treatments to induce cross-sensitivity (13), chemical modification of existing antimicrobials, e.g., by conjugation to iron-chelating molecules (50, 51), as well as the use of phytochemicals. Colistin adjuvants (including EDTA targeting metal ion chelation) have demonstrated synergistic effectiveness *in vitro* and *in vivo* in bacteremia in experimental animals (52). Furthermore, polymyxin derivatives with reduced cytotoxicity and antibacterial activity, such as polymyxin B nanopeptides, have been shown to potentiate antibiotics, such as aztreonam (53). While phytochemicals, such as epoxytiglianes, exhibit relatively low direct antimicrobial activity, their ability to interact with the outer membrane and dysregulate QS-signaling affords plants considerable fitness advantages against bacteria and bacterial biofilm colonization (54). Although numerous studies have described phytochemicals, and indeed terpenes (e.g., thymol and cinnamaldehyde) in the potentiation of antibiotics, translation to the clinic is poor (19). This may relate to a range of factors, but also likely reflects poor efficacy against biofilm infections *in vivo*. A major advantage of the semi-synthetic diterpene EBC-1013 for the topical treatment of cutaneous infections is its ability to stimulate the resident dermal inflammasome and induce re-epithelialization via PKC activation (24, 55).

We show here the antimicrobial potential of the semi-synthetic epoxytigliane EBC-1013, and how it interacts with and modifies the physicochemical properties of the outer membrane and effectively potentiates the action of colistin against planktonic colistin-resistant bacteria and biofilm communities. EBC-1013 is highly lipophilic by nature and rapidly binds to membranes close to the site of administration. Previous *in vivo* studies with EBC-1013 administered topically in bovine and murine wound models (24) showed no evidence of either local or systemic toxicities at therapeutically relevant concentrations. EBC-1013 is currently in a human clinical phase I dose-escalation safety study evaluating treatment side effects and systemic responses in patients with unresponsive antibiotic-resistant infections in venous leg ulcers (Australian New Zealand Clinical Trials Register: ACTRN12624000544572).

## MATERIALS AND METHODS

### Bacterial strains, media, and antimicrobials

The strains used in this study are detailed in Table S1. For colistin-resistant CX-17 strains, conjugation experiments were performed using a liquid mating method and wild-type donors. Overnight cultures of donor strains and CX-17 (recipient) were diluted 1:100 and incubated at 37°C (170 rpm) for 2 h to reach the exponential growth phase (equivalent to an optical density OD_600_ of 0.4). Donors and recipients were then mixed in a 1:3 ratio and incubated statically (37°C; 16–20 h). Colistin was added at final concentrations of 0.01 mg/L. Mating cultures were then serially diluted in sterile saline and plated onto selective agar plates containing 2 µg/mL colistin and nalidixic acid (16 µg/mL; to select for plasmid-carrying CX-17 transconjugants). The *mcr*-positive transconjugants were confirmed by PCR of *mcr-1/mcr-3* and *E. coli* phylogenetic group. Colistin resistance in the strains was validated for levels above those of EUCAST breakpoints using Clinical and Laboratory Standards Institute guidelines for MIC (56). Bacterial colonies were grown on Luria-Bertani broth (LB) agar (Lab M), and overnight cultures were grown in LB broth (Lab M) at 37°C, shaking, unless otherwise stated.

The naturally occurring EBC-147 and the semi-synthetic EBC-1013 epoxytigliane compounds (supplied by QBiotics Group Ltd.) were prepared at stock concentrations of 20 mg/mL in ethanol. Vehicle (ethanol equivalent) controls were included for each experiment. Colistin sulfate was purchased from Sigma-Aldrich Ltd. (Dorset, UK).

### Antimicrobial susceptibility testing

Conventional broth microdilution MIC assays of EBC-1013 and EBC-147, along with colistin and vehicle-only controls, were conducted in line with standard guidelines (57) using cation-adjusted Mueller-Hinton (MH) broth (LabM).

Checkerboard assays were conducted by serially diluting a two-dimensional array of serial concentrations of two test compounds (epoxytiglianes against colistin) at a range of concentrations as previously described (58, 59). The combination therapies were assessed from the calculated FIC index, which was determined as the inhibitory concentration of the combination therapy divided by that of the single antibiotic or epoxytigliane (60). The FIC index was determined from the lowest concentration of the combination of antibiotic and epoxytigliane, permitting no visible bacterial growth.

### Molecular dynamics simulations

The membrane built for MD simulations represents the *E. coli* K12 laboratory strain with an upper membrane containing R1 core-Lipid A (ECLPA) molecules with branching K12 core sugars and no O-antigen units and was created based on previously published reference membranes (61) using the CHARMM-GUI web interface (62). The simulation was then run in a box with epoxytiglianes and colistin added in various combinations (Table S4). The simulations were performed in triplicate, with each repeat being taken from a new minimized structure with a unique randomly generated starting set of velocities. Each repeat first underwent steepest descent minimization with a tolerance of 1,000 KJ/nm. The membrane then underwent six stages of equilibrium prior to the production run. For all equilibrium simulations, the Berendsen temperature coupling thermostat was applied, with Nose-Hoover used for the production run. Particle-mesh Ewald was applied to long-range electrostatics, and the simulations were run in NPT ensemble at a temperature of 310.15 K. The production run was for a total of 300 n.s. Images were produced using PyMOL.

### X-ray photoelectron spectroscopy

*E. coli* HRS.18 and CX-17 O/N cultures were cultivated in 150 mL LB broth with or without 1 μg/mL colistin at 37°C for 17 h at 120 rpm shaking. Cells from the O/N cultures were collected by centrifugation at 4,000 rpm for 10 min at 4°C, followed by two washes in phosphate-buffered saline (PBS). The optical densities were then adjusted to OD_600_ 1.1 in PBS. Subsequently, 10 mL of the adjusted bacterial suspension was transferred to a new Universal tube, centrifuged at 4,000 rpm for 10 min at 4°C, and resuspended and incubated in colistin, EBC-1013, or a combined treatment of the two at 37°C for 1 h. For HRS.18, colistin and EBC-1013 were used at 2 and 128 μg/mL, respectively, while for CX-17, they were used at 0.5 and 128 μg/mL. After incubation, treated cells were harvested and washed once using PBS; they were then dropped directly onto the cooling stage of the spectrometer and cooled in the load lock to below 250 K before exposure to vacuum.

XPS data were acquired on a Kratos Axis SUPRA using monochromated Al kα (1486.69 eV) X-rays at 15 mA emission and 12 kV HT (180 W) and a spot size/analysis area of 700 × 300 µm. The instrument was calibrated to gold metal Au 4f (83.95 eV), and dispersion adjustment gave a BE of 932.6 eV for the Cu 2p3/2 line of metallic copper. Ag 3d_5/2_ line FWHM at 10 eV pass energy was 0.544 eV. Resolution with charge compensation system on <1.33 eV FWHM on PTFE. High-resolution spectra were obtained using a pass energy of 20 eV, a step size of 0.1 eV, and a sweep time of 60 s, resulting in a line width of 0.696 eV for Au 4f_7/2_. Survey spectra were obtained using a pass energy of 160 eV. Charge neutralization was achieved using an electron flood gun with filament current = 0.4 A, charge balance = 2 V, filament bias = 4.2 V. Successful neutralization was adjudged by analyzing the C1s region, wherein a sharp peak with no lower BE structure was obtained. Spectra were charge corrected to the main line of the carbon 1s spectrum (adventitious carbon) set to 284.8 eV. Sample cooling was achieved using a ceramic contact cooled using flowing nitrogen, submerged in liquid nitrogen, and controlled using a Eurotherm temperature controller. All data were recorded at a base pressure of below 9 × 10^−9^ Torr and a room temperature of 294 K. Data were analyzed using CasaXPS v2.3.19PR1.0 (63). Peaks were fit with a Shirley background prior to component analysis. Comparison of the carbon spectra for colistin and EBC-1013 with the combined protein and lipid signals reported by the UMEÅ group (64, 65) revealed sufficient differences to permit deconvolution. Analysis of the carbon spectra was employed to deconvolute the carbon signals as lipid, polysaccharide, and protein. Models for colistin and EBC were derived from spectra of these molecules adsorbed on inert silica (S). The models were implemented in CASAXPS and are available in the open-access supplementary data files (Table S5; File S1).

### Hydrophobicity assay

Overnight cultures of *E. coli* J53, *E. coli* J53(pE30), and *E. coli* J53(pWJ1) in MH broth were standardized to 0.5 McFarland standard prior to treatment for 1 h (37°, 120 rpm) with colistin (½ MIC), epoxytiglianes (256 μg/mL), or vehicle controls. The samples were then centrifuged (3,000 × *g*, 5 min), washed (×2), and resuspended in Milli-Q water (66). Bacterial lawns were prepared by filtering 10 mL of bacterial suspension through 0.22 µm pore size Durapore polyvinylidene difluoride membrane filters (PVDF, Millipore) and air-dried in a laminar flow cabinet for 15 min. Samples were then mounted onto glass slides with double-sided adhesive tape and further air-dried for 5 min. Contact angle measurements of Milli-Q water (5 μL) were made using the sessile drop method with an Attension Theta Lite contact meter (Biolin Scientific). Contact angles (20 images/s) were obtained immediately after deposition of the drop on the *E. coli* lawns (or filter only controls) and recorded over a 10 s interval (*n* ≥ 3).

### Permeability assay

Cell permeability of *E. coli* J53, *E. coli* J53 (pE30), and *E. coli* J53 (pWJ1) was determined using the SYTOX Green Nucleic Acid Stain (Thermo Fisher Scientific) as previously described (67). EBC-1013 was tested at a concentration range of 128, 256, and 512 μg/mL, while colistin treatment was tested in the concentration range of ½ MIC to fourfold MIC to determine the lowest concentration of colistin that did not permeabilize the cell (Fig. S3). Combination treatments with this colistin concentration and EBC-1013 were also tested. An untreated control, vehicle control, and positive control (70% [vol/vol] isopropanol; IPA) were also included.

### Transmission electron microscopy

Overnight cultures of *E. coli* HRS.18 were filtered through a polyvinylidene difluoride membrane filter (PVDF, Durapore Membrane Filter, hydrophilic) of 0.22 μm pore size (Sigma-Aldrich Ltd., Dorset, UK) under negative pressure (20 mm Hg) for cell collection. The filtered cells were washed from the membrane filter in PBS. The resultant bacterial suspension was adjusted to 1.5 at OD_600nm_ in PBS and treated with 2 μg/mL colistin, 128 μg/mL EBC-1013, or a combination of 2 μg/mL colistin and 128 μg/mL EBC-1013 at 37°C for 1 h. An ethanol equivalent (vol/vol) at 128 μg/mL was also tested as a negative control. Thereafter, cells were fixed in 2.5% glutaraldehyde for 2 h at room temperature. Fixed cells were collected by filtration onto 0.45 mm nucleopore membrane filters (Camlab, Cambridge, UK), gently scraped off, resuspended in an equal volume of 4% low-melting-point agarose (ThermoFisher Scientific, Newport, Wales, UK) at 50°C, allowed to cool, and cut into 1 mm^3^ blocks. Blocks were post-fixed in 2% aqueous osmium tetroxide for 2 h, thoroughly washed in deionized water, and block-stained with 2% aqueous uranium acetate for 2 h. Following thorough washing in deionized water, samples were dehydrated through graded IPA (50%, and 70% for 10 min each, 100% for 2 × 15 min), infiltrated with hard grade TAAB embedding resin (TER) (TAAB Laboratories Equipment Ltd., UK) (50% in IPA for 30 min, and 4 × 1 h in pure resin) and embedded in TER at 60°C for 24 h.

Thick sections of 0.35 mm were cut on an Ultracut E ultramicrotome, dried onto glass slides, stained with 1% toluidine blue in 1% sodium tetraborate, mounted in Gurr’s neutral mountant, and examined with an Olympus BX51 research light microscope (Olympus Optical Co. Ltd., London, UK). Suitable areas were identified for subsequent ultrathin sectioning, and 80–100 nm thick sections were cut, collected onto 300 mesh copper grids, and stained with Reynolds lead citrate. Samples were examined in a Hitachi HT7800 TEM (Hitachi High Tech Ltd., UK) at 100 kV and images captured with Radius software (EMSIS GmbH, Germany).

### Biofilm disruption assays

Biofilm disruption assays were conducted as previously described (24). Briefly, established 18 h biofilms were treated for a further 24 h ± 128 µg/mL EBC-1013 or colistin (*E. coli* CX-17; 8 μg/mL and *E. coli* CX-17(pPN16); 32 μg/mL), along with combination treatment and a vehicle control (vol/vol). Biofilms were stained with 0.5% LIVE/DEAD BacLight stain (Invitrogen) in PBS before CLSM imaging (*n* = 3). CLSM was performed using a Zeiss LSM980 at ×63 magnification under oil. CLSM images were acquired using the same high-resolution parameters of 1,024 × 1,024 pixels, with a z-step size of 0.7 µm. The resultant z-stack images were analyzed by COMSTAT software (68) to produce measurements of biofilm biomass and DEAD/LIVE ratio as previously described (47).

### Statistical analysis

The FIC index was presented as the median of the calculated FIC indices. Group-wise comparisons for hydrophobicity and cell permeabilization assays were analyzed by parametric one-way ANOVA with Dunnett’s multiple comparison tests, with *P* ≤0.05 considered significant. Group-wise comparisons for CLSM COMSTAT analysis were undertaken using parametric one-way ANOVA with Dunnett’s multiple comparison tests, or non-parametric Kruskal-Wallis tests to ensure normality of the data sets. Graph Pad Prism (GraphPad Software Inc., La Jolla, USA) was used for all statistical analyses.

## Data Availability

All data associated with this study are present in the paper or the Supplementary Materials. The MD simulation data are available on Zenodo (https://doi.org/10.5281/zenodo.17232412).

## SUPPLEMENTAL MATERIAL

Supplemental Material mbio.02314-25-s0001.docx Supplemental tables and figures.

Raw Data mbio.02314-25-s0002.xlsx Raw data for figures.

ASM does not own the copyrights to Supplemental Material that may be linked to, or accessed through, an article. The authors have granted ASM a non-exclusive, world-wide license to publish the Supplemental Material files. Please contact the corresponding author directly for reuse.
