# Supplementary material for: Epoxytiglianes potentiate the activity of colistin against resistant Escherichia coli via modification of the bacterial cell membrane
Source: mBio. 2025 Dec 29;17(2):e02314-25. doi: 10.1128/mbio.02314-25 (PMC12892952; doi:10.1128/mbio.02314-25)
Supplement: Supplemental Material — Supplemental tables and figures. [file mbio.02314-25-s0001.docx]

**Epoxytiglianes potentiate the activity of colistin against resistant *Escherichia coli* via modification of the bacterial cell membrane**

Manon F. Pritchard,^1^ Wenya Xue,^1^ Jingxiang Wu,^1,2^ Francesca Boardman,^1^ Mei Li,^3^ Yuqing Zhou,^3^ Saira Khan,^1^ Lydia C. Powell,^1,4^ Joana Stokniene,^5^ Josh Davies-Jones^6^, Philip R. Davies^6^, Niklaas J. Buurma^7^, Georgina E. Menzies,^8^ Owen B. Spiller,^9^ Timothy R. Walsh,^3^ Paul Reddell,^5^ Katja E. Hill,^1^ & David W. Thomas^1^

**SUPPLEMENTARY MATERIAL**

**FIG S1** Molecular dynamic simulations images showing the beginning, middle and end point of the simulations shown in Fig 1. Colistin is shown in black, EBC-1013 in blue and EBC-147 in purple. The *E. coli* membrane is shown in red and grey, and the white spheres are circulating ions.

**FIG S2** Determination of hydrophobicity. Contact angle (°) measurements (A) at the 0 time point and (B) mean contact angle over 0-10 seconds for *E. coli* bacterial lawns treated with epoxytiglianes (256 μg/ml) alongside untreated, colistin (½ MIC; 0.032 or 2 μg/ml) and vehicle (256 μg/ml equivalent) controls. Contact angle measurements of < 90° are indicative of a hydrophilic surface. *Significantly different from untreated control (P < 0.05).

**FIG S3** Cell permeabilization assay showing the effect of colistin treatment (at given concentration) on colistin-sensitive CX-17 and colistin-resistant *E. coli*. Untreated (negative) and 70% isopropanol (IPA; positive) controls were also included. Results are expressed as fluorescence intensity (A.U.). *represents significantly different compared to the untreated control (n=3; P < 0.05).

**TABLE S1** Characteristics of colistin-sensitive (COL^Sens^) and colistin-resistant (COL^R^; *mcr*) Enterobacteriaceae farm isolates used in this study.

**TABLE S2** X-ray photoelectron spectroscopy (XPS) screening to show the effects of the incorporation or attachment of both colistin and EBC-1013 to the bacterial outer membrane of the colistin-sensitive CX-17 and *mcr* HRS-18 *E. coli* strains by region expressed as % of C (carbon) signal.

**TABLE S3** Statistical significance from CLSM Comstat analysis of *E. coli* CX-17 and CX-17(pPN16) biofilms treated with EBC-1013, colistin or both.

**TABLE S4** Molecular dynamics (MD) simulations were run in a box with epoxytiglianes and colistin added in various combinations as shown below.

**TABLE S5** Summary of the peak parameters used to create the models for the peptides, lipids, polysaccharides, colistin and EBC-1013 for fitting the XP spectra.


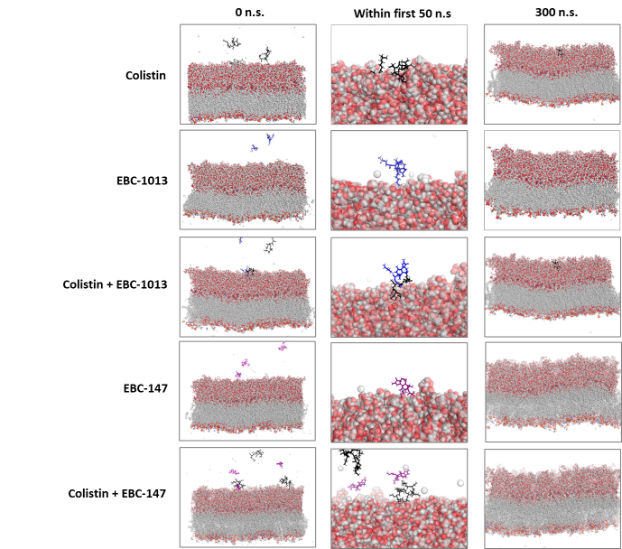


**FIG S1** Molecular dynamic simulations images showing the beginning, middle and end point of the simulations shown in Fig 1. Colistin is shown in black, EBC-1013 in blue and EBC-147 in purple. The *E. coli* membrane is shown in red and grey, and the white spheres are circulating ions.


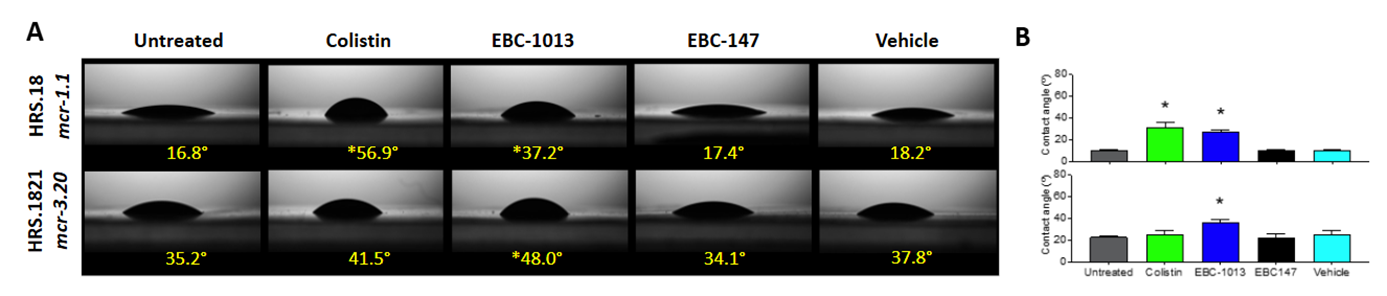


**FIG S2** Determination of hydrophobicity. Contact angle (°) measurements (A) at the 0 time point and (B) mean contact angle over 0-10 seconds for *E. coli* bacterial lawns treated with epoxytiglianes (256 μg/ml) alongside untreated, colistin (½ MIC; 0.032 or 2 μg/ml) and vehicle (256 μg/ml equivalent) controls. Contact angle measurements of < 90° are indicative of a hydrophilic surface.

*Significantly different from untreated control (P < 0.05).


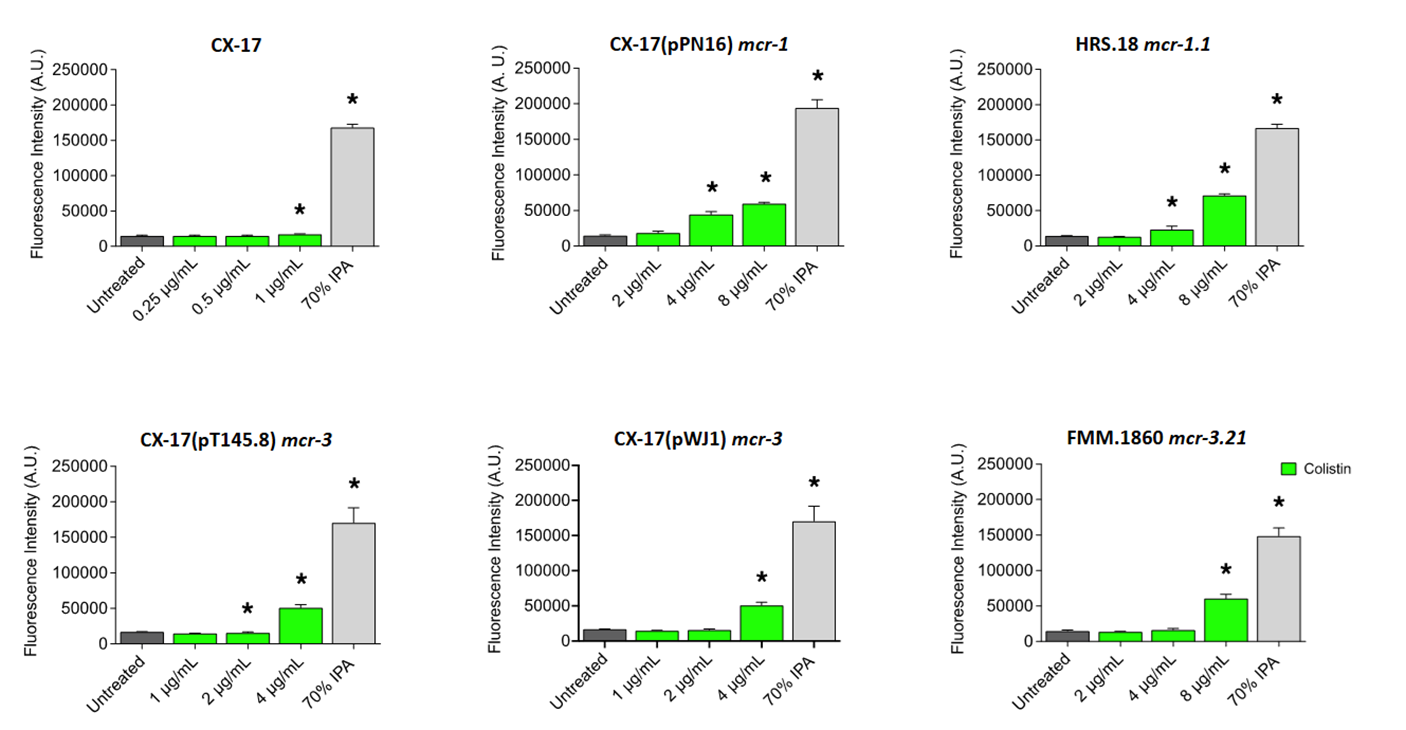


**FIG S3** Cell permeabilization assay showing the effect of colistin treatment (at given concentration) on colistin-sensitive CX-17 and colistin-resistant *E. coli*. Untreated (negative) and 70% isopropanol (IPA; positive) controls were also included. Results are expressed as fluorescence intensity (A.U.). *represents significantly different compared to the untreated control (n=3; P < 0.05).

**TABLE S1** Characteristics of colistin-sensitive (COL^Sens^) and colistin-resistant (COL^R^; *mcr*) Enterobacteriaceae farm isolates used in this study.

| **Strain ID^a^** | **Colistin Resistance** | **Incompatibility Group** | **Sequence Type (ST)** | **Source** | **Country of Origin** | **Reference** |
| --- | --- | --- | --- | --- | --- | --- |
| CX-17^c^ |  |  | 1193 | Animal | China | (1) |
| CX-17(pPN16)^b^ | *mcr-1* | Incl2 |  | Chicken meat | Thailand | This study |
| CX-17(pT145.4)^b^ | *mcr-1* | IncX4 |  | Swine faeces | Thailand | This study |
| CX-17(pWJ1)^b^ | *mcr-3* | IncHI2 |  | Swine faeces | China | (2, 3) |
| CX-17(pT145.8)^b^ | *mcr-3* | IncY |  | Swine faeces | Thailand | This study |
| HRS.18^c^ | *mcr-1.1* |  | 1081 | Human Rectal Swab | Laos | (1) |
| HRS.1821^c^ | *mcr-3.20* |  | 46 | Human Rectal Swab | Laos | (1) |
| FMM.1860^c^ | *mcr-3.21* | IncFII | 58 | Fly | Laos | (1) |
| NNMR 49.b^c^ | *mcr-1* |  |  | Human Rectal Swab | Nigeria | This study |

^a^All *Escherichia coli* strains except NNMR 49.b (*Klebsiella pneumoniae*).

^b^Transconjugants derived from transfer of mobile plasmid-encoded COL^R^ (*mcr*) from an original wild-type host into CX-17.

^c^Wildtype farm isolates.

1. Zhou, Y. Farzana, R. Sihalath, S. Rattanavong, S. Vongsouvath, M. Mayxay, M. Sands, K. Newton, P. N. Dance, D. A. B. Hassan, B. & Walsh, T. R. A one-health sampling strategy to explore the dissemination and relationship between colistin resistance in human, animal, and environmental sectors in Laos. Engineering. 15, 45-56, (2022).
2. Yin, W. Li, H. Shen, Y. Liu, Z. Wang, S. Shen, Z. Zhang, R. Walsh, T. R. Shen, J. & Wang, Y. Novel plasmid-mediated colistin resistance gene mcr-3 in Escherichia coli. MBio 8, e00543-17 (2017).
3. Li, M. 2023. Assessment of the global impact of MCR-1/MCR-3 mediated colistin resistance. PhD thesis. Cardiff University, UK.

**TABLE S2** X-ray photoelectron spectroscopy (XPS) screening to show the effects of the incorporation or attachment of both colistin and EBC-1013 to the bacterial outer membrane of the colistin-sensitive CX-17 and *mcr* HRS-18 *E. coli* strains by region expressed as % of C (carbon) signal.

|  |  | **% of C (carbon) signal** | | | | | | |
| --- | --- | --- | --- | --- | --- | --- | --- | --- |
| **Strain** | **Sample** | **C** | **N** | **O** | **P** | **S** | **Cl** | **Na1s** |
| CX-17 | Untreated | 100 | 13.8 | 42.9 | 5.6 | 0.5 | 2.4 | 2.2 |
|  | Colistin | 100 | 15.0 | 31.6 | 2.7 | 0.8 | 1.9 | 1.3 |
|  | EBC-1013 | 100 | 7.2 | 24.9 | 1.9 | 0.3 | 4.0 | 1.3 |
|  | Colistin + EBC-1013 | 100 | 5.4 | 27.6 | 2.8 | 0.0 | 0.9 | 1.1 |
| HRS.18 | Control | 100 | 13.1 | 35.4 | 3.3 | 0.5 | 3.6 | 1.6 |
|  | Colistin | 100 | 11.6 | 41.6 | 2.9 | 0.7 | 3.2 | 2.1 |
|  | EBC-1013 | 100 | 4.4 | 24.0 | 2.6 | 0.1 | 1.2 | 0.6 |
|  | Colistin + EBC | 100 | 8.2 | 27.4 | 1.4 | 0.2 | 3.6 | 0.9 |
|  | Colistin measured | 100 | 59.1 | 32.5 |  |  |  |  |
|  | Colistin expected | 100 | 30.8 | 25.0 |  |  |  |  |
|  | EBC-1013 measured | 100 | 0.0 | 35.3 |  |  |  |  |
|  | EBC-1013 expected | 100 |  | 31.0 |  |  |  |  |

**TABLE S3** Statistical significance (*P* values) from CLSM Comstat analysis of *E. coli* CX-17 and CX-17(pPN16) biofilms treated with EBC-1013, colistin or both.

|  |  |  | **Treatment** | | | |
| --- | --- | --- | --- | --- | --- | --- |
| **Strain** | **Comstat Analysis** | **Treatment** | **Vehicle** | **Colistin** | **EBC-1013** | **EBC-1013 + Colistin** |
| CX-17 | Biomass Volume | Untreated | **0.0199** | **<0.0001** | **0.0298** | **<0.0001** |
|  |  | Vehicle |  | **<0.0001** | >0.9999 | **<0.0001** |
|  |  | Colistin |  |  | **<0.0001** | 0.9989 |
|  |  | EBC-1013 |  |  |  | **<0.0001** |
|  | Dead/Live Cell Ratio | Untreated | **0.0072** | **0.0278** | 0.3246 | >0.9999 |
|  |  | Vehicle |  | **<0.0001** | >0.9999 | **0.0002** |
|  |  | Colistin |  |  | **<0.0001** | 0.3712 |
|  |  | EBC-1013 |  |  |  | **0.0221** |
| CX-17(pPN16) | Biomass Volume | Untreated | 0.3549 | 0.1120 | >0.9999 | **<0.0001** |
|  |  | Vehicle |  | 0.9956 | 0.9659 | **<0.0001** |
|  |  | Colistin |  |  | 0.7282 | **0.0053** |
|  |  | EBC-1013 |  |  |  | **0.0050** |
|  | Dead/Live Cell Ratio | Untreated | >0.9999 | >0.9999 | 0.7433 | >0.9999 |
|  |  | Vehicle |  | >0.9999 | 0.0043** | >0.9999 |
|  |  | Colistin |  |  | 0.2830 | >0.9999 |
|  |  | EBC-1013 |  |  |  | 0.9455 |

Values in bold denote significance

**TABLE S4** Molecular dynamics (MD) simulations were run in a box with epoxytiglianes and colistin added in various combinations as shown below.

| **Compound** | **Time** | **Repeats** |
| --- | --- | --- |
| Colistin (3 molecules) | 300 ns | 3 |
| EBC-147 (3 molecules) | 300 ns | 3 |
| EBC -1013 (3 molecules) | 300 ns | 3 |
| Colistin + EBC 147 (3 + 2 molecules) | 300 ns | 3 |
| Colistin + EBC 1013 (3 + 2 molecules) | 300 ns | 3 |

**Table S5.** Summary of the peak parameters used to create the models for the peptides, lipids, polysaccharides, colistin and EBC-1013 for fitting the XP spectra.

| Model name | Component name | Position /eV | Relative Conc. |
| --- | --- | --- | --- |
| Peptide | C1 | 284.99* | 1 |
|  | C1 | 286.2 | 0.5 |
|  | C1 | 288.25 | 0.5 |
|  |  |  |  |
| Lipid | C2 | 284.99* | 1 |
|  | C2 | 285.83 | 0.14 |
|  | C2 | 287.1 | 0.07 |
|  | C2 | 289 | 0.07 |
|  |  |  |  |
| Polysaccharide | C3 | 284.81 | 0.04 |
|  | C3 | 286.65^$^ | 1 |
|  | C3 | 287.88 | 0.24 |
|  | C3 | 289 | 0.03 |
|  |  |  |  |
| Colistin model | CHx | 284.79* | 1 |
|  | Colistin-OH/NH | 286.2 | 0.44 |
|  | Colistin CON | 288.06 | 0.23 |
|  | Colistin NH | 399.89 | N |
|  | Colistin NH2 | 401.48 | 0.48*N |
|  | Colistin C=O | 531.16 | O |
|  | Colistin OH | 533.21 | 0.24*O |
|  | Silica from support | 532.12 | Variable |
|  |  |  |  |
| EBC-1013 model | CHx | 284.78* | 1 |
|  | EBC C-OH | 286.58 | 0.17 |
|  | EBC CO2 | 289.13 | 0.03 |
|  | EBC-C=O/OH | 532.24 | O |
|  | EBC-CO2 | 533.24 | 0.18*O |

*Main peak constrained to within +/- 0.25 eV of 285 eV. All other C(1s) peaks constrained to fixed positions relative to main peak.
